# Caffeine Inhibits Direct and Indirect Angiogenesis in Zebrafish Embryos

**DOI:** 10.3390/ijms22094856

**Published:** 2021-05-03

**Authors:** Ram Manohar Basnet, Daniela Zizioli, Alessia Muscò, Dario Finazzi, Sandra Sigala, Elisa Rossini, Chiara Tobia, Jessica Guerra, Marco Presta, Maurizio Memo

**Affiliations:** 1 Unit of Pharmacology, DMMT, University of Brescia, 25123 Brescia, Italy; rmb.basnet@gmail.com (R.M.B.); a.musco@unibs.it (A.M.); sandra.sigala@unibs.it (S.S.); e.rossini013@unibs.it (E.R.); 2 Unit of Biotechnology, DMMT, University of Brescia, 25123 Brescia, Italy; daniela.zizioli@unibs.it (D.Z.); dario.finazzi@unibs.it (D.F.); 3 Laboratorio Centrale Analisi Chimico-Cliniche, ASST Spedali Civili, 25123 Brescia, Italy; 4 Unit of Experimental Oncology and Immunology, DMMT, University of Brescia, 25123 Brescia, Italy; chiara.tobia@unibs.it (C.T.); j.guerra@unibs.it (J.G.); marco.presta@unibs.it (M.P.)

**Keywords:** caffeine, angiogenesis, FGF2, zebrafish, methylxanthines, embryonic vascular development

## Abstract

In this study, we report the effects of caffeine on angiogenesis in zebrafish embryos both during normal development and after exposure to Fibroblast Growth Factor 2 (FGF2). As markers of angiogenesis, we measured the length and width of intersegmental vessels (ISVs), performed whole-mount in situ hybridization with *fli1* and *cadh5* vascular markers, and counted the number of interconnecting vessels (ICVs) in sub-intestinal venous plexus (SIVP). In addition, we measured angiogenesis after performing zebrafish yolk membrane (ZFYM) assay with microinjection of fibroblast growth factor 2 (FGF2) and perivitelline tumor xenograft assay with microinjection of tumorigenic FGF2-overexpressing endothelial (FGF2-T-MAE) cells. The results showed that caffeine treatment causes a shortening and thinning of ISVs along with a decreased expression of the vascular marker genes and a decrease in the number of ICVs in the SIVP. Caffeine was also able to block angiogenesis induced by exogenous FGF2 or FGF2-producing cells. Overall, our results are suggestive of the inhibitory effect of caffeine in both direct and indirect angiogenesis.

## 1. Introduction

Angiogenesis is the formation of new blood vessels. This process is a significant component of a wide variety of physiological processes, including embryonic vascular development, differentiation, wound healing and organ regeneration, and pathological processes, including tumor progression, rheumatoid arthritis, and psoriasis [1]. Growing evidence suggests that vascular perturbation and angiogenesis play also a critical role in the pathogenesis of Alzheimer’s disease [2,3]. Knowing the fine modulatory pathways regulating angiogenesis is thus fundamental for developing novel therapeutic strategies [4].

Zebrafish embryos are emerging as an in vivo model for the study of angiogenesis. The anatomy of the developing vascular tree, the angiogenesis process, and the molecular mechanisms involved in vascular structure formation are strikingly similar to those found in humans and higher vertebrates [5]. Besides the general advantages, zebrafish possess angiogenesis-specific advantages compared to other animal models. The optical transparency of the zebrafish embryos makes it easy to directly visualize and track the changes in the developing vascular system [5,6]. Moreover, the development of different transgenic reporter lines specific for vascular markers provides further tools to study the angiogenic process [7]. 

The study of angiogenesis in zebrafish embryos usually relies on the investigation of two vasculature structures: the intersegmental vessels (ISVs) and the sub-intestinal venous plexus (SIVP). ISVs are part of the trunk vasculature and run between the somites. Their formation begins at around 22 h post-fertilization (hpf) and represents the first angiogenetic process easily visualized [5]. The formation of SIVP takes place in both the left and right dorsolateral sides of the yolk. It starts as early as 30 hpf with the migration of cells from the posterior cardinal vein (PCV), and it is completed by 72 hpf with the formation of a complete SIV basket connected with intercapillary branches. Changes in the formation of this structure represent a typical readout for treatments with pro or anti-angiogenic effects [8,9]. Recently, experimental approaches, such as zebrafish yolk membrane (ZFYM) angiogenesis assay and tumor xenotransplantation (TX) assays [10,11], have been developed to exploit the potential of SIVP for the study of the impact of small molecules and growth factors on developmental vascularization. Angiogenic inhibitors can be broadly classified into direct and indirect ones. Direct inhibitors may act directly on endothelial cells, whereas indirect inhibitors suppress the activity of growth factors such as vascular endothelial growth factor (VEGF) and fibroblast growth factors (FGFs) [12]. Caffeine is one of the widely used psychostimulants in the world. It is present in commonly consumed beverages, such as coffee, tea, and energy drinks [13]. Caffeine acts primarily by adenosine receptor antagonism and phosphodiesterase inhibition [14,15]. Experimental evidence indicates that caffeine may act as a direct angiogenesis inhibitor. For instance, caffeine has been shown to inhibit developmental angiogenesis in zebrafish embryos via a direct effect on endothelial cells [16]. In addition, caffeine reduces the levels of expression of VEGF, one of the most relevant growth factors involved in angiogenic signaling, in the chick embryo chorioallantoic membrane [17,18] and in zebrafish larvae [19]. These data are consistent with the observation that caffeine inhibits VEGF expression in tumor cells via an adenosine receptor antagonist mechanism of action [20,21]. In addition, in keeping with a direct effect on endothelial cells, caffeine induces apoptosis in human umbilical vein endothelial cells (HUVECs) [17]. In contrast with these observations, caffeine has been proposed to stimulate HUVEC migration and morphogenesis by altering mithocondrial distribution and energetics [22]. Finally, caffeine has been shown to decrease LPS-induced nitric oxide production in zebrafish embryos, suggesting the possibility that caffeine may also exert anti-angiogenic effects via an indirect mechanism of action following the suppression of pro-inflammatory responses [23]. Thus, the role of caffeine as a direct and/or indirect angiogenesis inhibitor deserves further investigation. On this basis, in this study, we investigated the effect exerted in vivo by caffeine on direct and indirect angiogenesis in zebrafish embryos. For the first aim, we studied the development of intersegmental vessels (ISVs) and sub-intestinal vascular plexus (SIVP) in embryos exposed to different doses of caffeine. In a second set of experiments, we assessed the effect of caffeine on indirect angiogenesis as evaluated in the ZFYM and TX angiogenesis assays. The results demonstrate that caffeine exerts a significant inhibitory effect on both direct and indirect angiogenesis, underlying the potential impact of caffeine on physiological and pathological angiogenesis.

## 2. Results

### 2.1. Caffeine Inhibits Developmental Angiogenesis in Zebrafish Embryos

To study the effect of caffeine in developmental angiogenesis, we focused on the development of ISVs, modeling of caudal venous plexus (CVP) and formation of SIVP, together with the analysis of the expression of the vascular markers *fli1* and *cadh5* at different developmental stages.

#### 2.1.1. Effect of Caffeine on the Formation of ISVs

**Figure 1:**
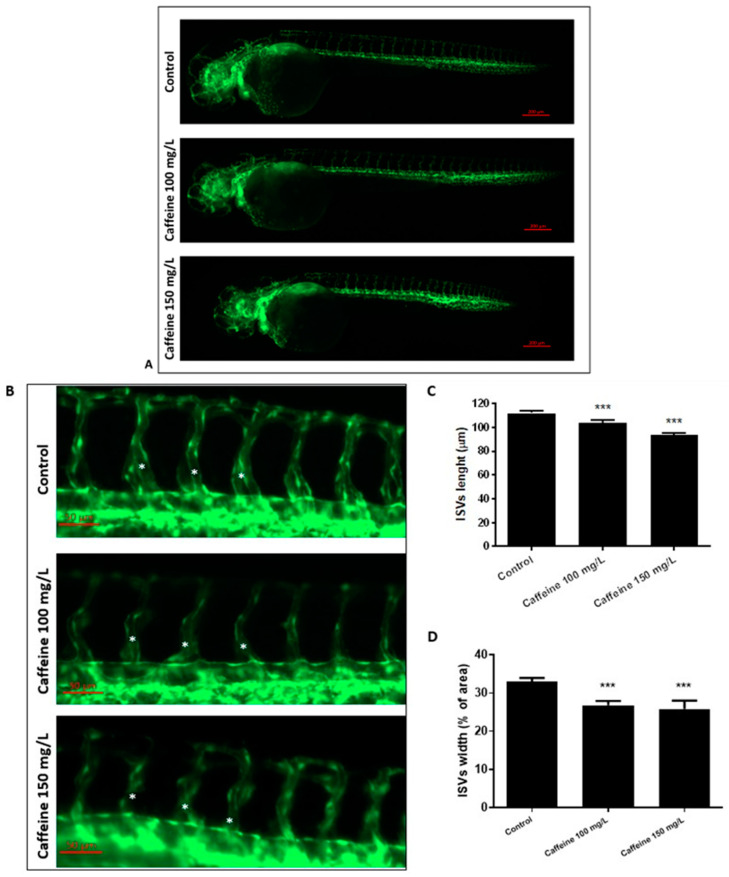
Caffeine affects ISVs’ development. (**A**) Representative, lateral-view pictures of Tg(kdrl:EGFP) control and caffeine-treated embryos at 48 hpf. Magnification 32×. (**B**) Enlargement of the tail of embryos shown in A. Magnification 63×. White asterisks point to the ISVs considered for the measurement. (**C**,**D**). The graphs represent the mean values obtained for ISVs length and width measurements, respectively, both in control and treated embryos. Three replicates were performed, with 10 embryos per group. *** *p* < 0.0005.

We measured the length and width of ISVs in embryos exposed to 100 and 150 mg/L of caffeine from 15 until 48 hpf. Embryos raised in sterile water were used as control. Caffeine-treated embryos had significantly reduced length and width of ISVs (Figure 1) as compared to the controls. The reduction of the length and width of ISVs did not involve the adjoining somites, as shown by phalloidin staining (Figure S1, Supplementary Material). As shown in Figure S2, the effect of caffeine induces a permanent disruption in angiogenesis. 

#### 2.1.2. Caffeine Prevents the Remodeling of CVP

**Figure 2:**
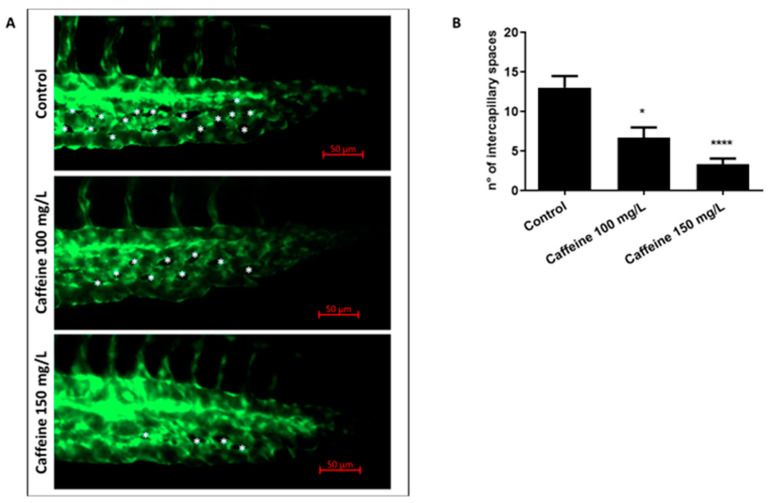
Caffeine affects the number of intercapillary spaces in the CVP. (**A**) Representative pictures showing the CVP of Tg(kdrl:EGFP) control and treated embryos at 48 hpf. Magnification 32×. Asterisks point to the intercapillary spaces. (**B**) The bar diagram in the panel shows the average number of intercapillary spaces in control and caffeine (100 and 150 mg/L)-treated embryos. Three biological replicates were performed, with 10 embryos per group. Statistical analysis was performed with GraphPad Prism software, version 8.3.0 (GraphPad Software, Inc., La Jolla, CA, USA). * *p* < 0.05, **** *p* < 0.0005, when compared with the control group.

We also counted the number of intercapillary spaces in the CVP of zebrafish embryos. CVP forms a dense capillary network in the tail of the embryos. For the proper formation of this structure, capillaries should be able to remodel themselves, thereby preventing fusion of the blood vessels and forming a distinct capillary network [24]. The presence of intercapillary spaces in the CVP indicates a proper remodeling of the blood vessels. As shown in Figure 2, embryos treated with caffeine had a significantly reduced number of intercapillary spaces, as compared to the controls. We counted 13 ± 1.4 intercapillary spaces in control embryos, and 6 ± 1.3 and 3 ± 0.6 intercapillary spaces in embryos exposed to 100 or 150 mg/L of caffeine, respectively. 

#### 2.1.3. Caffeine Reduces the Expression of Vascular Markers *fli1* and *cadh5*

**Figure 3:**
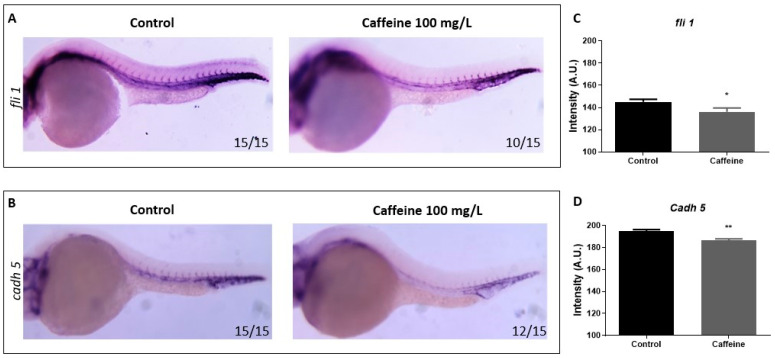
Caffeine-treated embryos display a reduced expression of *fli1* and *cad5*. Representative lateral views of the WISH for *fli1* (**A**) and *cadh5* (**B**) probes, performed in 28 hpf embryos raised in sterile water (control) or treated with caffeine 100 mg/L. Magnification 32×. Ratios at the bottom right part of each picture specify the number of embryos showing the same staining pattern, compared to the total number of embryos used for each experiment (n = 15). (**C**,**D**) The bar diagrams show the quantification of the colorimetric signal in control and caffeine-treated embryos. The expression of *fli1* and *cadh5* was significantly reduced in the caffeine-treated embryos. For *cadh5*, two replicates were performed (n = 15), while for *fli1*, the experiments were conducted in triplicate (n = 15). * *p* < 0.05, ** *p* < 0.005, when compared with control group.

*fli1* is a gene belonging to the ETS family and it plays a key role as an upstream transcription regulator in angiogenesis [25]. *cadh5* is an endothelial-specific transmembrane protein that clusters at adherens junctions, where it promotes homotypic cell–cell adhesion [26]. We observed a shortening and thinning of the ISVs in caffeine-treated embryos. To further investigate this phenotype, we performed whole-mount in situ hybridization (WISH) in 28 hpf embryos with *fli1* and *cadh5* as the specific vascular markers. The comparison of the hybridization pattern of the probes in control and caffeine (100 mg/L)-treated embryos at 28 hpf did not show a significant perturbation in the spatial distribution of *fli1* and *cadh5* expression, but a reduction in the intensity of the colorimetric signal in the treated embryos (Figure 3). The quantification of the signal intensity assessed by ImageJ analysis confirmed significant differences among groups (Figure 3C,D). The results are indicative of a general down-regulation of genes involved in endothelial differentiation (*fli1*) and structural integrity of blood trunk vessels (*cadh5*).

#### 2.1.4. Caffeine Inhibits the Formation of SIVP

**Figure 4:**
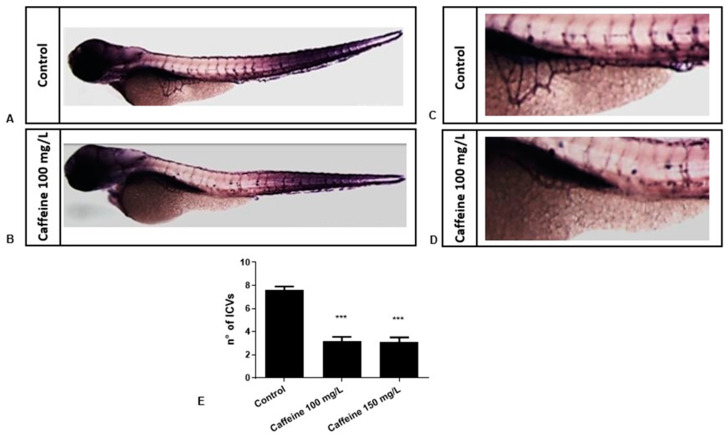
Caffeine exposure decreases the number of interconnecting vessels in the SIVP. (**A**,**B**) The panels show representative pictures of AP assay performed in control and treated embryos at 72 hpf (magnification 32×), with an enlargement of the SIVP (magnification 63×) in **C** and **D**. (**E**) The bar diagram shows the average number of SIV branches in zebrafish embryos at 72 hpf (three replicates were performed, n = 15 for each group). Statistical analysis was performed with GraphPad Prism software**.** *** *p* < 0.001, when compared with the control group.

To further investigate the role of caffeine in angiogenesis, we explored its effect in the development of SIVP. We exposed zebrafish embryos to the two doses of the drug from 15 until 48 hpf. At 48 hpf, the treatment solution was removed and the embryos were raised in sterile water until 72 hpf. At 72 hpf, embryos were fixed in 4% (*v*/*v*) PFA and whole-mount Alkaline Phosphatase (AP) staining was done to visualize the development of SIVP. The number of interconnecting branches was counted for the treated and control embryos. We found a significant reduction in the number of ICVs in the caffeine-treated embryos at both doses, as compared to the controls (Figure 4). In addition, we observed that the size of the SIVP basket was smaller in the caffeine-treated embryos (45/50), as compared to the controls.

### 2.2. Caffeine Inhibits FGF2 Mediated Angiogenesis

After observing the inhibitory effect of caffeine in developmental angiogenesis, we wanted to know if caffeine could block angiogenesis mediated by external proangiogenic stimuli. Therefore, we used FGF2 and FGF2-overexpressing cells as a proangiogenic stimulus and performed zebrafish yolk membrane (ZFYM) and tumor xenograft (TX) assays, respectively.

#### 2.2.1. Caffeine Inhibits FGF2 Induced Neo-Angiogenesis

**Figure 5:**
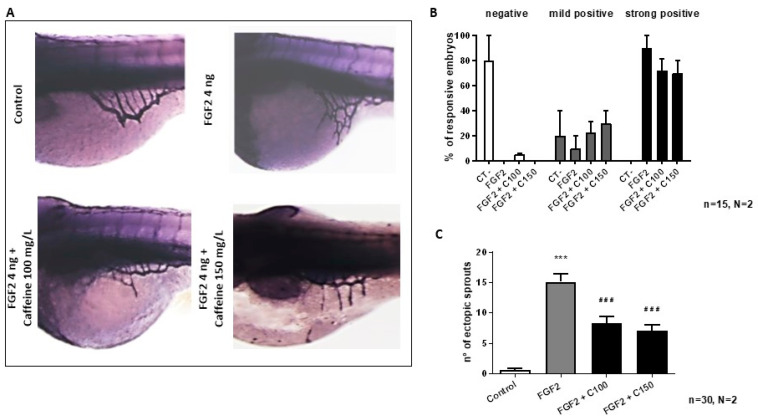
Evaluation of the effects of caffeine treatment on FGF2-microinjected embryos. (**A**) Representative picture of 72 hpf embryos after AP assay (magnification 63×). (**B**) Percentage of embryos showing negative, mild positive, and strong positive response following microinjection of FGF2, with or without caffeine treatment. Caffeine treatment in the FGF2 microinjected embryos did not show a significant decrease in either strong positive or mild positive response. However, analysis of the number of ectopic sprouts showed that caffeine treatment significantly reduced the number of ectopic sprouts in the FGF2-injected zebrafish embryos (**C**). In the bar diagrams: CT-: negative control; FGF2 + C100: FGF2 + caffeine 100 mg/L; FGF2 + C150: FGF2 + caffeine 150 mg/L. Statistical analysis was performed with GraphPad Prism software, version 8.3.0 (GraphPad Software, Inc., La Jolla, CA, USA). *** *p* < 0.001 between control and FGF2, ### *p* < 0.001 between FGF2 and caffeine treated groups (FGF2 + C100 and FGF2 + C150).

To perform ZFYM assay, zebrafish embryos were injected with 4 ng of FGF2 into the perivitelline space, close to the developing SIVP at 48 hpf. After the injection, embryos were raised in sterile water or treated with 100 or 150 mg/L caffeine until 72 hpf and then fixed in 4% (*v*/*v*) PFA and stained with AP assay, as described in the Section Materials and Methods and in Supplementary Figure S3. In normal conditions, injection of FGF2 stimulates the formation of ectopic sprouts from the SIV basket (Figure 5A) [10]. As described in Section Materials and Methods and Supplementary Figure S4, we distinguished two types of positive response: the mild and the strong one. The lack of sprouts induction was classified as a negative response.

Control embryos showed about 15 sprouts, whereas treated embryos had only a mean of 8 and 6 sprouts for the low and high dose, respectively (Figure 5C). 

However, there was no difference in the number of strongly positive embryos in the caffeine-treated and control embryos (Figure 5B). 

#### 2.2.2. Caffeine Inhibits Neo-Angiogenesis Induced by Tumorigenic Cells 

**Figure 6:**
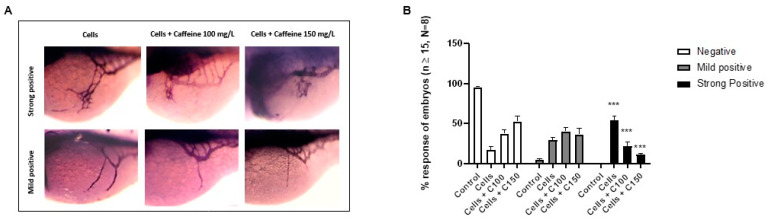
Microinjection of 3F2T cells in perivitelline space and subsequent treatment with caffeine. (**A**) Representative picture showing the ectopic sprouts branching out of SIVP visualized by AP assay in microinjected embryos at 72 hpf embryos (magnification 63×). (**B**) Percentage of embryos showing negative, mild positive and strong positive responses following microinjection of 3F2T cells, with or without caffeine treatment. In the bar diagram: CT-: negative control; Cells + C100: Cells + caffeine 100 mg/L; Cells + C150: Cells + caffeine 150 mg/L. Statistical analysis was performed with GraphPad Prism software, version 8.3.0 (GraphPad Software, Inc., La Jolla, CA, USA). *** *p* < 0.001, when compared to the control group.

To further explore the effect of caffeine in neo-angiogenesis, we performed zebrafish TX assay by injecting the 3F2T tumorigenic cells in proximity to the developing SIVP at 48 hpf. The injected embryos were immediately transferred to Petri dishes containing caffeine 100 or 150 mg/L. After 24 hpf, the embryos were fixed with 4% (*v*/*v*) PFA and stained with the AP assay for the visualization of the ectopic sprouts. We found that caffeine treatment significantly decreased the number of embryos with a strongly positive angiogenic response, as compared to the control (Figure 6B). For negative, mild positive, and strong positive response classification, the same criteria described in the previous section for FGF2 ectopic sprouts induction were used. 

## 3. Discussion

Angiogenesis plays critical roles in human physiology and pathology that range from fetal growth to tissue repair and cancer [4]. Thus, numerous therapeutic opportunities can be envisaged through the successful understanding and subsequent manipulation of angiogenesis. In this study, we investigated the antiangiogenic effects of caffeine by exploiting various angiogenesis assays in zebrafish embryos. The results demonstrate that caffeine is able to block both developmental and FGF2-mediated angiogenesis. The inhibition of developmental angiogenesis resulted in a decrease in length and width of ISVs, decreased number of ICVs in SIVP, and reduced expression of vascular markers *fli1* and *cadh5*. Similarly, the inhibition of FGF2-mediated angiogenesis was demonstrated by the ZFYM and zebrafish xenograft assays in which caffeine was able to reduce angiogenesis induced by proangiogenic stimuli, represented by recombinant FGF2 protein and tumorigenic cells overexpressing FGF2. In a previous study, caffeine was shown to perturb angiogenesis during zebrafish development [16]. Our results confirm these observations and extend the spectrum of caffeine activity, including inhibition of FGF2-mediated angiogenesis. 

The mechanism(s) of action responsible for the inhibitory effects on angiogenesis induced by caffeine is (are) still unclear. Methylxanthines, including caffeine, are known to act via adenosine receptor antagonism and phosphodiesterase inhibition [14,15], and both actions have been implicated in the antiangiogenic pathways. It has been shown that caffeine inhibits growth factors involved in angiogenesis in different cellular models [16,17,20]. In addition, evidence is available showing that other methylxanthines, such as pentoxifylline [27] and theophylline [28], possess antiangiogenic properties. Pharmacological studies with adenosine receptor agonists and antagonists may help in elucidating the functional target(s) of caffeine responsible for its capacity to inhibit angiogenesis.

Angiogenesis inhibitors are classified into direct inhibitors and indirect inhibitors [12]. Direct inhibitors target endothelial cells in the growing vasculature and include drugs such as angiostatin, endostatin, and arrestin. Indirect inhibitors block the activity of angiogenesis inducers. As evidenced by our findings, caffeine can be included in the list of drugs acting as both direct and indirect inhibitors of angiogenesis, thus unraveling novel pharmacological applications for this class of compounds.

## 4. Materials and Methods

### 4.1. Zebrafish Maintenance and Collection of Eggs

Zebrafish were maintained and used in accordance with the Italian and European rules on animal use following protocols approved by the local committee (OPBA) and authorized by the Ministry of Health (Authorization Number 393/2017). Adult AB wild type and transgenic line Tg (*kdrl*:EGFP) [29] zebrafish lines were raised and maintained at 28 ℃ in 14 h light and 10 h dark cycle in a circulating system maintained at pH 7.0–7.5, and conductivity between 400–500 µs. Fish were fed with a combination of granular food (from Special Diet Services, SDS, Witham, UK) and freshly prepared *Artemia* sp. (cysts bought from SDS, Witham, UK). Adult male and female zebrafish were put at the breeding tanks overnight, so that freshly spawned eggs could be collected the next morning. Unfertilized or dead eggs were removed. Then, these were raised in fish water (0.1 g/L Instant Ocean Sea Salts, 0.1 g/L Sodium Bicarbonate, 0.19 g/L Calcium Sulfate) with incubation at 28.5 °C until the experiments. The staging of zebrafish embryos was done as described in Kimmel et al. [30]. Embryos at 24 h post-fertilization were treated with 0.003% 1-phenyl-2-thiourea (PTU) to prevent pigmentation. 

### 4.2. Caffeine Treatment

Fresh solution of caffeine was prepared on the day of the experiment by dissolving caffeine powder (Sigma-Aldrich, St. Louis, MO, USA) in sterile water (vehicle). Two different doses, 100 and 150 mg/L of caffeine, were selected based on our previous study where static treatment with 150 mg/L or less caffeine did not induce lethality in zebrafish embryos exposed from 3 to 72 hpf [31]. Caffeine exposure to zebrafish embryos was done by immersion method: embryos at the desired stage were placed in a 100 mm × 15 mm Petri dish containing either caffeine solution (treated) or the vehicle (control) and incubated in static condition (no daily solution renewal). For the analysis of ISVs, CVP, and SIVP, the wild type and transgenic embryos Tg(*kdrl*:EGFP) were treated from 15 until 48 hpf. For the ZFYM and TX assays, wild type zebrafish embryos were exposed to caffeine from 48 until 72 hpf (Supplementary Figure S2).

### 4.3. Cell Culture 

FGF2-overexpressing murine aortic endothelial cells (FGF2-T-MAE cells) were maintained in Dulbecco’s modified minimum essential medium (DMEM, Life Technologies Waltham, MA, USA) supplemented with 10 mM Hepes (Life Technologies), 10% (*v*/*v*) fetal bovine serum (FBS), 1 mM sodium pyruvate (Life Technologies), and 500 μg/mL geneticin (Invitrogen, Waltham, MA, USA). Cells were maintained in a humidified incubator at 37 °C in the presence of 5% CO₂ and split 1:16 weekly. Cells were periodically tested for mycoplasma. 

### 4.4. Image Analysis of ISVs and CVP

Measurement of length and width of ISVs was performed in Tg(*kdrl*:EGFP) embryos treated or not treated with caffeine. Dead embryos and embryos showing toxicity such as pericardial edema were excluded from the experiments. At 48 hpf, fluorescent images depicting the vascular tree were acquired for the control and treated groups. The images were taken in lateral position at 32× magnification with Zeiss Axiozoom V13 (Zeiss, Jena, Germany) fluorescence microscope, equipped with PlanNeoFluar Z 1×/0.25 FWD 56 mm lens and Zen pro software.

The images were then processed, and the length and width of ISVs were measured with Image J Fiji software (Rasband, W.S., ImageJ, U. S. National Institutes of Health, Bethesda, MD, USA). Two landmarks, one dorsal and one ventral, were established to define the section of ISVs to be measured: ventrally, the commencement of the ISV at the upper border of dorsal aorta; dorsally, the bifurcation of ISVs into dorsal longitudinal anastomotic vessels (DLAVs).

To measure the length of ISVs, 10 embryos per group were analyzed. For each embryo, three intersegmental vessels corresponding to the yolk sac extension were selected. 

The width of ISVs was determined following the quantification method described by Hans et al. [32]. A region of interest (ROI) enclosing 5 ISVs corresponding to the yolk sac extension, was selected for each embryo. The ROI was then converted to greyscale and thresholded to obtain the bidimensional trace of ISVs over a background hull. The width of each ISV was determined as normalized ISV area, calculated by dividing the area of each ISV trace by the area given by the background. 

The same images were also utilized to manually count the number of intercapillary spaces in the CVP of control and treated embryos [24]. 

### 4.5. WISH 

Wild-type zebrafish embryos were raised with or without 100 mg/L caffeine at 15 hpf. At 28 hpf, 20 embryos without any obvious deformities were selected for each condition and fixed with 4% (*v*/*v*) paraformaldehyde (PFA), dehydrated in 100% (*v*/*v*) methanol, and stored at −20 °C. WISH was performed with vascular probes: VE-Cadherin (*cadh5*) and *fli1*. The preparation of the probes and WISH in zebrafish embryos was performed as previously described [33]. WISH images were taken with a Leica MZ16F stereomicroscope equipped with DFC 480 digital camera and LAS Leica Imaging software (Leica Microsystems, Wetzlar, Germany) at 63× magnification.

WISH pictures were quantified with Image J Fiji software as follows. The region of the embryo tail containing the WISH signal (approximately from the mid-yolk region to the tip of the tail) was selected, and the intensity was measured. An equal area of the tail outside of the stained area was selected to determine the background. The value of the colorimetric signal was then obtained by subtracting the background from the measured intensity.

### 4.6. AP Staining

AP assay was performed in order to visualize the SIVP in zebrafish embryos after treatment with caffeine and the ectopic sprouts formation in SIVP after ZFYM and TX assays. Wild-type zebrafish embryos (n = 50) were raised with or without caffeine 100 and 150 mg/L at 15 hpf. At 24 hpf, 0.003% PTU was added to the embryos to prevent pigmentation. The embryos were evaluated for any mortality and toxicity every 24 h. Any embryos that were dead or showing toxic effects (abnormal phenotype) were removed. Embryos with pericardial edema and tail abnormality (hyperextension, flexion, lateral bending) were considered as abnormal phenotype. At 72 hpf, embryos from each condition and control groups were fixed in 4% (*v*/*v*) PFA. AP assay was performed as described elsewhere [34]. Briefly, embryos were put in 100% (*v*/*v*) methanol-phosphate-buffered saline (PBS) solution. The embryos were then equilibrated in Tris buffer (100 mM Tris HCl pH 9.5, 50 mM MgCl₂, 100 mM NaCl, 0.1% Tween-20) and stained with nitro-blue tetrazolium chloride (NBT) and 5-bromo-4-chloro-3’-indolyphosphate p-toluidine salt (BCIP) solution. The stained SIVP was evaluated under Leica stereomicroscope at 60× magnification. In particular, the ICVs in the SIVP were manually counted for the control and caffeine-treated embryos. The ICVs at the left side of the embryos were counted as left the SIVP develops and matures before the right [8].

### 4.7. ZFYM Assay

ZFYM assay was performed as described elsewhere [10]. AB wild-type zebrafish embryos were raised until 48 hpf in fish water with 0.003% PTU. At 48 hpf, the embryos were manually dechorionated using forceps and anesthetized in 0.04 mg/L tricaine. Embryos were then placed in the lateral position in a Petri dish coated with 0.1% agarose. Borosilicate needles were loaded with FGF2 (1 mg/mL) and mounted in the microinjector. The embryos were injected with 4 nL of FGF2 under the guidance of Leica stereomicroscope at 60× magnification. After microinjection, the embryos were immediately treated with caffeine (100 and 150 mg/L). Embryos injected with PBS 1× (140 mM NaCl, 10 mM phosphate, 3 mM KCl; pH 7.4) were used as negative control. The rest of the embryos were raised in sterile water. All the embryos were incubated at 28.5 °C for the next 24 h. At 72 hpf, the embryos were fixed in 4% (*v*/*v*) PFA, and AP assay was performed to evaluate the blood vessel formation (Supplementary Figure S3). Injection of FGF2 induced the formation of ectopic sprouts around the SIVP. In order to evaluate if caffeine prevents angiogenesis, the number of ectopic sprouts branching out from the SIVP was manually counted on embryos injected with FGF2 and FGF2, with or without caffeine (100 and 150 mg/L). Controls were the embryos injected with PBS (pH 7.4). The ectopic sprouts were counted on both sides of the embryos under Leica Stereomicroscope at 63× magnification (Supplementary Figure S4). It was possible to distinguish two types of responses directly induced by FGF2 injection: mild positive responses were characterized by the appearance of 2–3 sprouts, while the formation of a larger number of sprouts was considered a strong positive response. Furthermore, the embryos showing a dense network of ectopic sprouts in which it was difficult to distinguish each of the sprouts were considered strong responders.

### 4.8. Phalloidin Staining

Phalloidin staining of zebrafish embryos was performed as follows. Manually dechorionated embryos were fixed in 4% (*v*/*v*) PFA for 3 h at room temperature (RT). Then, embryos were washed three times in PBS (1× (140 mM NaCl, 10 mM phosphate, 3 mM KCl; pH 7.4)/0.1% Tween 20 (PBST) for 10 min and incubated in blocking solution (10% goat serum, 2% BSA, 0.5% Triton X-100 in PBS) for 2 h at RT. Embryos were incubated for 3 h at RT with Alexa Fluor 594 Phalloidin (1:400 in blocking solution, Thermo Fisher Scientific, Waltham, MA, USA). Unbound antibody was removed by several PBST washes.

### 4.9. Zebrafish TX Assay

AB wild-type zebrafish embryos were raised until 48 hpf in fish water with PTU. At 48 hpf, the embryos were dechorionated and anesthetized in 0.04 mg/L tricaine. Then, 4 nL of 3F2T cells with concentration of 500,000 per mL were injected into the perivitelline space close to the developing SIVP as described elsewhere [35]. After microinjection, embryos were immediately transferred to the caffeine (100 and 150 mg/L) solution for the treatment group and sterile water for the control group. The embryos were then incubated at 33 °C, the suggested temperature for mammalian cells-injected fish [36]. At 72 hpf, the embryos were fixed in 4% (*v*/*v*) PFA, and AP assay was performed as described before to evaluate the angiogenesis.

### 4.10. Statistical Analysis

The data were collected for the control group and the treated group. Each experiment was repeated a minimum of two times. The data are presented as mean ± SEM. The significance test of all the data was analyzed with either Student *t*-test or one-way analysis of variance (one-way ANOVA). *p*-value less than 0.05 was considered significant: * *p* < 0.05, ** *p* < 0.005, *** *p* < 0.0005.

## Data Availability

Data are available from the corresponding author upon request.

## Supplementary Materials

Supplementary materials can be found at https://www.mdpi.com/article/10.3390/ijms22094856/s1.
