# Supplementary figures and images for: Caffeine Inhibits Direct and Indirect Angiogenesis in Zebrafish Embryos
Source: Int J Mol Sci. 2021 May 3;22(9):4856. doi: 10.3390/ijms22094856 (PMC8124397; doi:10.3390/ijms22094856)

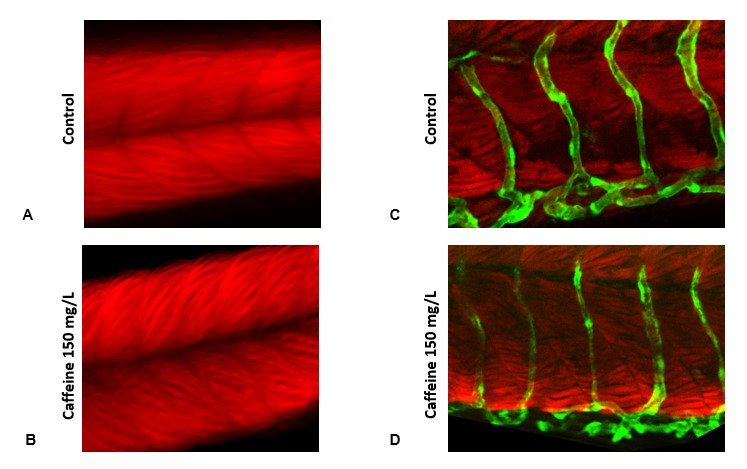

Supplement: Supplementary file 1 [file ijms-22-04856-s001.zip › Figure S1.tiff]

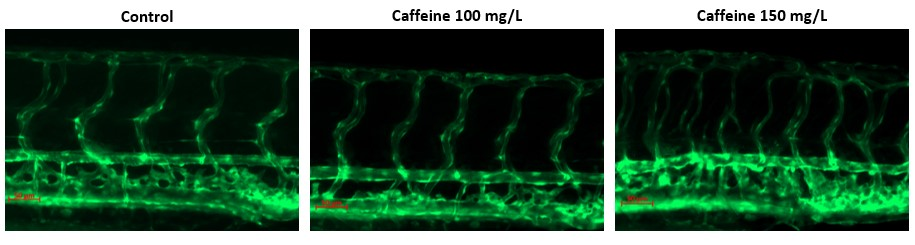

Supplement: Supplementary file 1 [file ijms-22-04856-s001.zip › Figure S2.tiff]

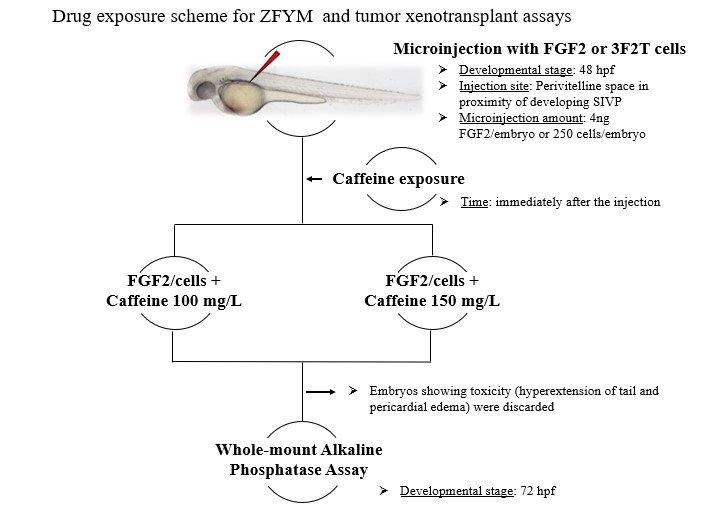

Supplement: Supplementary file 1 [file ijms-22-04856-s001.zip › Figure S3.tiff]

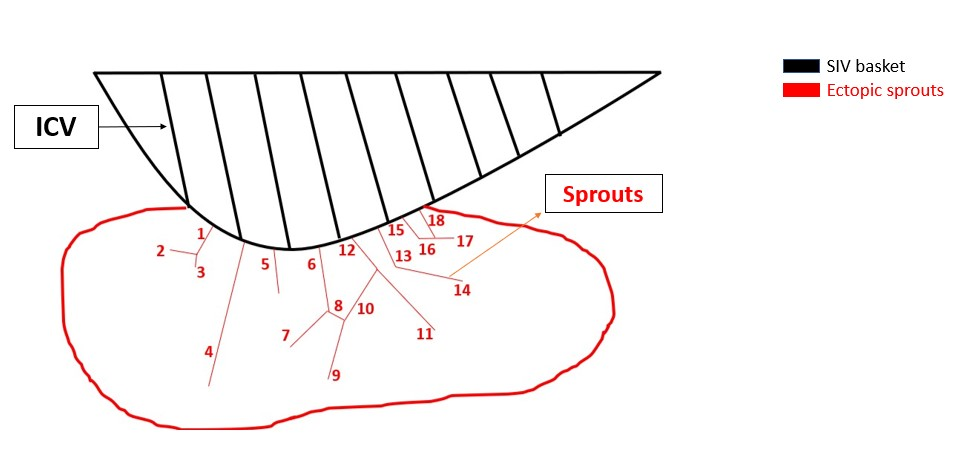

Supplement: Supplementary file 1 [file ijms-22-04856-s001.zip › Figure S4.tiff]
